# Assessing health system factors affecting access and delivery of IPTp-SP and ITN to pregnant women attending ANC clinics in Ghana

**DOI:** 10.1186/s12913-021-07055-2

**Published:** 2021-10-06

**Authors:** Virtue Fiawokome De-Gaulle, Pascal Magnussen, Joseph Kamgno, Wilfred Mbacham, Verner N. Orish, Harry Tagbor

**Affiliations:** 1 Faculty of Medicine and Biomedical Sciences, University of Yaoundé I, Yaoundé, Cameroon; 2 Faculty of Health and Medical Sciences, University of Copenhagen, Copenhagen, Denmark; 3 School of Medicine, University of Health and Allied Science, Ho, Ghana

**Keywords:** Health system, Intermittent preventive treatment, Insecticide treated nets, Pregnant woman, Antenatal clinic, Ghana

## Abstract

**Introduction:**

Malaria interventions including use of Sulfadoxine-Pyrimethamine as Intermittent Preventive Treatment (IPTp-SP) and distribution of Insecticide Treated Nets (ITNs) have been implemented through ante-natal clinic (ANC) services in Ghana. Yet, the high ANC attendance is not commensurate with the uptake of these interventions, with missed opportunities to deliver the interventions. This study sought to assess the health system factors affecting access and delivery of IPTp-SP and ITN as defined by the Ghana Malaria Policy Guideline to eligible pregnant women attending ANC clinic sessions.

**Methods:**

A quantitative cross-sectional study was conducted in the Volta Region of Ghana, with data collected across three levels of health care delivery facilities, including hospitals, health centres and Community-Based Health Planning Service (CHPS) compounds. Data collection included structured observation checklists to document the communication and interaction between the ANC health staff and pregnant women. Additionally, structured questionnaires were used to elicit information on cadre, trainings attended, knowledge and delivery practices of health workers on IPTp-SP and ITN. Stata 16 was used for data analysis, and a defined delivery algorithm was used to compute appropriate and inappropriate delivery practices, using the Ghana policy directive as a guide. Predictors of appropriate delivery were determined using logistic regression analysis.

**Results:**

Approximately 97% of the 680 ANC observations had complete information for analysis. Of these, 78% (511/657) were eligible for IPTp-SP after excluding women who have less than 16 weeks of gestation, G6PD deficient, malaria positive and have taken 5 doses of IPTp-SP prior to day of observation. Appropriate delivery of IPTp-SP was 76% (390/511). Despite the availability of SP, 15% (75/511) of all eligible women were not offered the medication and 37% (44/119) of inappropriate delivery was recorded during periods of stock out. ITNs were appropriately delivered to 59% (139) out of 237 eligible women. Thirty-two percent (77/237) of eligible women, mostly continuing ANC clients, were not given ITN despite stock availability.

**Conclusions:**

IPTp-SP was appropriately delivered to most of the eligible pregnant women compared to ITN. While stock out of both intervention could account for inappropriate delivery, despite stock availability, IPTp-SP and ITN were not delivered to some eligible women.

## Background

Malaria, an infectious parasitic disease, transmitted by mosquitoes continues to be a grave public health problem for the African continent [1]. In 2019, 94% (215 million) of all malaria cases and deaths (384,000) were recorded on the continent, with most of the infections due to *Plasmodium falciparum*, the most lethal of the parasites [2]. Although everybody is at risk of the disease in endemic regions, children under 5 years old and pregnant women are the most vulnerable [3].

For pregnant women, their increased vulnerability has been attributed to reduced immunity arising from immunological, hormonal and physiological changes [4, 5]. For instance, Lindsay and colleagues reported that pregnant women compared to their non-pregnant counterparts were more readily detectable by mosquitoes at close range [6]. In their study, they found that the abdomen of each pregnant woman was averagely 0.7 °C hotter than that of non-pregnant women, and explained that this physiological change could facilitate an increased release of volatile substances from the skin surface and produce a larger host signature making it easy for mosquitoes to locate them [6]. In 2019, the World Health Organization (WHO) estimated that out of 33.2 million pregnancies that occurred in sub-Saharan Africa (SSA), approximately 11.6 million of them were exposed to malaria infection [3], and this resulted in about 822,000 low birth weighted (LBW) babies, half of whom were born in the West African sub region [2]. In Ghana, about 370,514 pregnancies are estimated to be exposed to malaria parasites annually, and this results in 30,611 LBW babies [7]. Malaria in pregnancy (MiP), mainly *P. falciparum* infection is associated with a number of adverse effects including maternal death, maternal anaemia, miscarriage, still birth, intra-uterine growth restriction and LBW [8–10]. The severity of the effects of the disease in pregnancy is however dependent on the level of acquired immunity [4]. In low or unstable malaria transmission areas, with low levels of acquired immunity, pregnant women are more likely to progress towards clinical cases, with untreated persons developing severe disease and even death [4, 5, 11]. In moderate and high transmission settings like most parts of SSA, malaria infection during pregnancy is predominantly asymptomatic due to acquired immunity, arising from recurrent infections [12, 13]. Nonetheless, the parasites may be present in the placenta and contribute to maternal anaemia [14, 15] which can lead to LBW [16, 17], an important contributor to infant mortality [18].

To mitigate the adverse effects associated with MiP, WHO developed a policy framework with a three pronged approach for malaria control among pregnant women in the African region [19]. This entailed [1] administering Intermittent Preventive Treatment using Sulfadoxine-Pyrimethamine (IPTp-SP), starting as early as possible in the second trimester, with monthly intervals for subsequent doses and delivered under Directly Observed Therapy (DOT) [2]; distribution and counselling on use of Insecticide Treated Nets (ITNs) [3]; with the third being Prompt Case Management of clinical malaria [19]. Evidence from a meta-analysis on higher mean birth weight and less placental malaria from the uptake of three or more IPTp-SP [20] led to WHO updating the policy framework by recommending at least three doses of IPTp-SP during pregnancy to ensure optimal protection, in addition to the other interventions of ITN use and prompt case management [21, 22]. These interventions have been incorporated into the ANC services, thus, IPTp-SP and ITN are given to eligible pregnant women during their scheduled ANC clinic visits [23]. Financial support from donor agencies including the Global Fund [24, 25] and local funding from governments of African countries have eased the financial burden on procurement of these preventive interventions, so that IPTp-SP and ITNs are to be given to pregnant women free of charge. With the implementation of these intervention at ANC clinics, it was expected that the high ANC attendance in most SSA countries would translate into comparable uptake of the interventions, resulting in substantial reduction in LBW babies. Unfortunately, this has not been the case, with huge disparities between ANC attendance and IPTp-SP uptake and ITN ownership recorded [26] and thousands of LBW babies attributable to malaria born in the region annually [3]. In 2016, the World Malaria Report (WMR) indicated that only 39% of pregnant women who attended ANC received an ITN [27], and in 2018, only 31% of pregnant women received IPTp-SP3 [3].

In Ghana, national estimates from the 2014 Demographic and Health Survey (GDHS) revealed that 87% of pregnant women attended four or more ANC visits, with most of them starting in their second trimester, but only 39% reported taking three or more doses of SP [28]. Whilst the uptake of these interventions could be influenced by individual factors such as pregnant woman’s acceptance or refusal to take the intervention based on personal, cultural or religious beliefs [29], health system factors directly linked to delivery of the interventions have been cited as a possible major cause of this disparity [24, 28]. Although some studies have been conducted in Ghana on IPTp-SP and ITN, they have been limited to assessing pregnant women factors influencing uptake [29–31]. Not much has been done on assessing the health system factors affecting delivery. of IPTp-SP and ITN. This study therefore sought to assess the delivery appropriateness of IPTp-SP and ITN to pregnant women attending ANC clinic sessions, using the Ghana MiP policy directive as a guide, and also ascertain health system factors influencing delivery of the two preventive interventions.

## Methods

### Study site

**Fig. 1:**
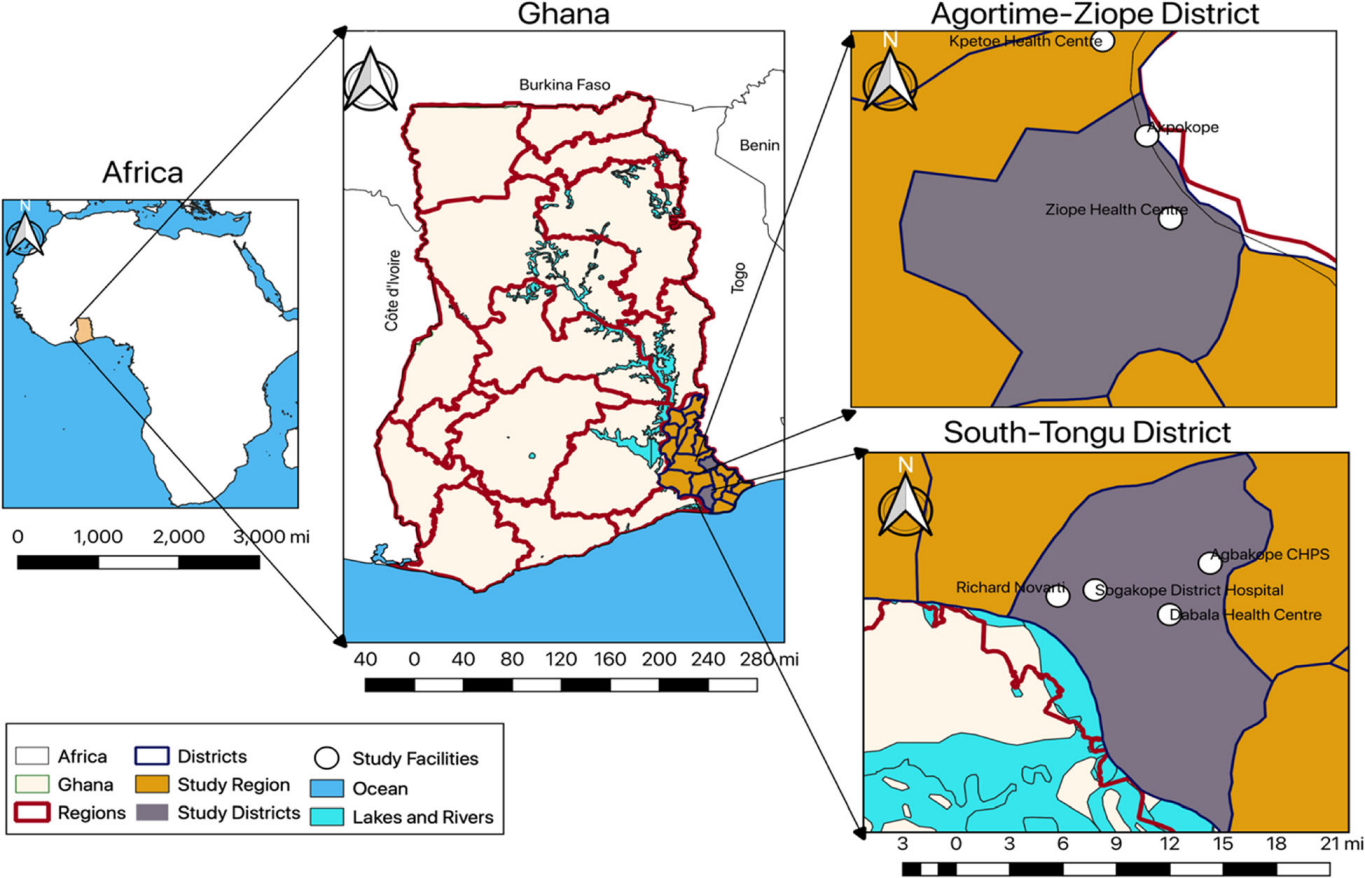
Study Site

This study was conducted in the Agortime-Ziope and South Tongu Districts located in the Volta Region of Ghana (Fig. 1). This research was part of a bigger study that sought to assess parasitic infections among pregnant women as well as an ethnographic study to ascertain the practices of pregnant women in preventing malaria. The study districts were therefore selected based on rural-urban characteristics of the settlements. The Agortime-Ziope district is predominantly rural, with a population of about 35,360, 49% (17,326) of whom are females [32]. Approximately, 39.4% (6843) of the females are between 15 and 49 years, and the General Fertility Rate (GFR), defined as the number of live births per 1000 women aged between 15 and 49 years in a given year was 109.2 according to the 2010 Census [32]. The district had no hospital at the time of the data collection, however, there were three health centres, four Community-Based Health Planning Services (CHPS) compound, and one private maternity home [32]. The South Tongu district is peri-urban with a population of 110,777, 52% of whom are females [33]. Approximately, 37% (21,325) of the females are aged between 15 and 49 years and the GFR (103.3) is comparable to that of Agortime-Ziope [33].. The district has a total of 29 health facilities (two hospitals, four health centres, 18 CHPS compounds and 5 private clinics) [33]. Data was collected across three levels of healthcare delivery facilities - hospitals, health centres and CHPS compound.

### The health care delivery system of Ghana

In Ghana, health care is provided by both public and private facilities with the Ministry of Health being the overarching body of governance [34]. The public health care services are facilities under the Ghana Health Service, Teaching Hospitals and Quasi-Government Institutions such as the Police Service, Military and Public Universities [34]. The private sector is made up of Faith-Based, Private-for-Profit, Private not-for-Profit health institutions and the Traditional Health System [34]. Health services are organized in a three-tier health delivery system of primary, secondary and tertiary level, with five levels of providers; CHPS compounds, health centers and clinics, district hospitals, regional hospitals and tertiary hospitals [34]. The CHPS compounds, health centers and clinics render primary health care, with the district hospitals rendering both primary and secondary health care and serving as the main referral hospital. The regional hospitals are the referral level for secondary care and they are run by general practitioners and specialists [34]. The teaching hospitals provide tertiary care and training of doctors [34]. To reduce out of pocket payments, Ghana instituted a National Health Insurance Scheme (NHIS) in 2003, financed through the National Health Insurance Fund (NHIF) which has three main funding sources; tax revenue from Value Added Tax (2.5%), contributions of Social Security and National Insurance Trust (2.5%) and income adjusted premiums ranging between seven Ghana Cedis to 48 Cedis [34]. A free maternal health care policy was also instituted in 2008, thus all pregnant women are entitled to free enrolment on the NHIS to enable access to maternal health services [35].

### Study design

This was a quantitative health facility-based cross-sectional study, with data collected over a period of 5 months; from April to June 2019 and from November to December, 2019. Seven health facilities across three levels of health care delivery system were included in this study. All the health facilities provided ANC services, however Focused ANC (FANC), which is an individualised, client centred, comprehensive care, with emphasis on disease detection rather than risk assessment [36] was limited to the hospitals and CHPS compounds. Although health centres had laboratory services, Glucose-6-phosphate dehydrogenase (G6PD) tests were conducted only in the hospital laboratories. MiP and case management guidelines were unavailable in some of the facilities, however, wall charts on IPTp-SP and ITN were visibly displayed on the walls in all the ANC units. Stock outs of SP (two health centres) and ITNs (one hospital and two health centres) were recorded during the data collection period. In the hospitals and health centres, a section outside the ANC consulting room was used for recording blood pressure, temperature, weight and height of the pregnant women. A waiting area was also designated for pregnant women to sit and wait their turn, with collective health education organized for them. Services provided to pregnant women in the ANC consulting room included history taking, physical examination (palpating the abdomen, measuring fundal height, and listening to foetal heartbeat), Point of Care (POC) tests (HIV tests, urine dipstick for protein and sugar, and malaria RDT), administration of treatments (IPTp-SP) and immunizations (Tetanus), case management of some disease conditions (e.g. uncomplicated malaria), distribution of ITN, and health education.

### Study population

The study population comprised of pregnant women and ANC health staff. Specifically, all pregnant women irrespective of gestational age attending ANC clinic during the study period were eligible to participate in the study. With regards to the ANC health staff, a complete enumeration of all ANC staff who provide clinical care in the study facilities was carried out.

### Sample size calculation

A sample size of 680 was calculated using Cochran’s formula (*n* = z^2^p (1-p)/e^2^) for calculating sample sizes for cross sectional studies. Where **“*n”*** is the computed sample size, **“*z”*** the desired confidence interval at 95% (z = 1.96)**, “*p”*** the estimated proportion of an attribute that is present in the population, and **“*e”*** the desired level of precision at 5% [37]. To determine the most appropriate prevalence **“p”** of the two interventions (IPTp-SP or ITN) to use, the most current available national estimates of IPTp-SP3 (36.7%) [38] and ITN use (43%) [39] at the time of the data collection period was used. ITN use generated the largest sample size of 377 for each study district (Table 1). The Cochrane’s finite population correction formula [37] was then used to correct the sample sizes for each district to reflect a feasible and representative number of pregnant woman that can be sampled, after which a 10% non-response or incomplete dataset rate was computed (Table 1). This percentage was informed by a Malawian study on IPTp-SP, where 8% of the data were incomplete, thus excluded from analysis [40]. The number of pregnant women who access ANC services in the various health facilities vary, therefore a proportionate to size was used to determine the percentage of pregnant women to be sampled in each facility in a particular district. This was determined by making the total number of annual ANC registrants (reference year is 2018 retrieved from the District Health Management Information system (DHIMS), in each health facility a fraction of the sum of all pregnant women registered for ANC in all the participating health facilities in each district annually. This fraction was then multiplied by the calculated sample size for each district to get the proportion of pregnant women to be sampled from each facility (Table 2).

**Table 1:** Sample Size Calculation

Uptake and use as “P”	**IPTp-SP3 (**36.7%**)**	**ITN Use (**43%**)**
$\boldsymbol{n}=\frac{{\boldsymbol{z}}^{\mathbf{2}}\boldsymbol{p}\left(\mathbf{1}-\boldsymbol{p}\right)}{{\boldsymbol{e}}^{\mathbf{2}}}$	357	377
Sample size for each district	**377**	
**Finite population correction for computed sample size**		
	**Agortime-Ziope**	**South-Tongu**
ANC Registrants for 2018 (N)	1078	3209
$n=\frac{n_0}{1+\frac{\left({n}_0-1\right)}{N}}$	280	338
**Adding a 10% non response rate**	**308**	**372**

**Table 2:** Proportionate to Facility Size Sample Calculation

Districts	Health facilities	Yearly, ANC registrants (N***i***)	Proportion of registrants wi = N***i*** / N	Sample per facility n***i*** = wi*n
South Tongu	Sogakope District Hospital	944	0.41	153
	Richard Novarti Catholic Hospital	931	0.41	151
	Dabala Health Centre	257	0.11	42
	Agbakope CHPS Compound	160	0.07	26
	**Total (N)**	**2292**	**1**	**372**
Agortime Ziope	Kpetoe Health Centre	544	0.58	180
	Ziope Health Centre	336	0.36	112
	Akpokope CHPS Compound	51	0.05	16
	**Total (N)**	**931**	**1**	**308**

### Data collection procedure

Data was collected in seven health facilities purposively sampled across three levels of care. This sampling was done to help identify the health system factors peculiar to each level of care, and ascertain how that affects the delivery of IPTp-SP and ITN. In the South Tongu District, the district hospital (Sogakope District Hospital), one health centre (Dabala Health Centre) and CHPS compound (Agbakope CHPS) with the highest number of ANC clients compared to the other CHPS and health centres in that district were included. Additionally, a faith-based facility (Richard Novarti Catholic Hospital (RNCH)) comparable to the level of a district hospital was also included to provide an insight into the delivery practices peculiar to non-governmental health facilities. In the Agortime-Ziope District, there was no district hospital at the time of data collection, two health centres; one in an urban (Kpetoe Health Centre) and the other in a peri-urban settlement (Ziope Health Centre) were included, in addition one CHPS compound (Akpokope CHPS) with the highest number of ANC clients was sampled. Data collection tools used included observation checklist and questionnaires. These tools were pre-tested in the Ho Polyclinic in the Volta region, with 10 ANC observations conducted and three ANC health workers interviewed with the questionnaires. Modifications including changing the wording for some questions for easy comprehension and adding other questions were effected. Two different observation checklists were used, one was used to document the interaction between ANC health staff and pregnant women and the second to document general ANC activities on a daily basis, including stock of IPTp-SP and ITN. Four community health nurses were recruited as research assistants (RAs) and trained on the study aim, data collection methods and research ethics for 2 days, followed by 1 day field work. The field work was to ensure that the RAs understood the data collection tools and also to make them conversant with the data collection process. Prior to commencing data collection, a meeting was convened in each study facility with the health workers to inform them about the study and also seek their consent to be observed, especially the ANC staff. Data collection commenced with all the consented ANC health staff interviewed with a questionnaire. The questionnaire elicited information on cadre, trainings attended and knowledge on the Ghana Policy on IPTp-SP and ITN. Consecutive sampling, a technique in which every study population meeting the inclusion criteria is sampled until the sampled size is reached [41] was employed in the sampling of pregnant women. The RAs approached pregnant women as they awaited their turn to be attended to, introduced the study to them and obtained written consent from those willing to participate. The observation of the ANC consultation sessions was then carried out, with the actions and communications between pregnant women and health staff documented. Data collected included the cadre of health worker delivering care, gestational age of pregnant woman, number of ANC sessions attended, physical examinations and tests conducted by the ANC staff, medications and treatments administered including IPTp-SP, and delivery of ITN. Data was collected on every ANC clinic day till the required sample size was obtained. All Filled data collection tools were cross-checked on a daily basis for data accuracy and completeness by a field supervisor.

### Study definitions

Using the 2014 Ghana MiP Policy directive [42], an algorithm to assess the delivery of IPTp-SP and ITN in the ANC unit was developed. The policy directive specified that SP should be administered as a single dose of three tablets of 500 mg Sulfadoxine and 25 mg pyrimethamine, commenced from 16 weeks of gestation or at quickening, to pregnant women who are not clinically diagnosed with malaria or G6PD deficiency. The medication should also be administered under Directly Observed Therapy (DOT) with monthly intervals. While, up to seven doses of SP can be taken as specified in the policy document, delivery in all the health facilities across Ghana has been capped at five doses. The defined algorithm for appropriate delivery of IPTp-SP for this study was therefore defined based on successful completion of three steps, which were:

i. Eligible pregnant women (≥16 weeks, not clinically ill with malaria, not G6PD deficient and taken <5doses of SP) being offered IPTp-SP by ANC health staff, and
ii. Administered under DOT and
iii. Informed her next ANC visit either verbally or written in their Maternal Health Record Book (MHRB) to foster continuity of the medication.

Inappropriate delivery of IPTp-SP entailed any of the actions or inactions below by the health worker:

i. Health worker not offering IPTp-SP to eligible pregnant women or,
ii. Health worker offering IPTp-SP, but not administering under DOT coupled with telling the pregnant women her next visit or not.
iii. Health worker offering IPTp-SP, under DOT, but not telling the pregnant women her next visit, verbally or writing it in her MHRB.

Similarly, delivery of ITN was assessed by an algorithm. In Ghana, every pregnant woman attending ANC clinic is eligible for an ITN free of charge. Hence, eligibility for ITN was defined as any pregnant woman who has not yet been given an ITN since starting ANC. Evidence of receipt of an ITN by a pregnant woman during her current pregnancy at the ANC is indicated through its documentation in the MHRB of the woman. Pregnant women with no documentation of ‘ITN given’ in their MHRB was interpreted as ITN not yet received. Appropriate delivery of ITN was therefore defined as:

i. Eligible pregnant women being given an ITN during ANC visit.

Inappropriate delivery was defined as:

i. Eligible pregnant woman not being given an ITN during ANC observation.

### Data analyses

**Fig. 2:**
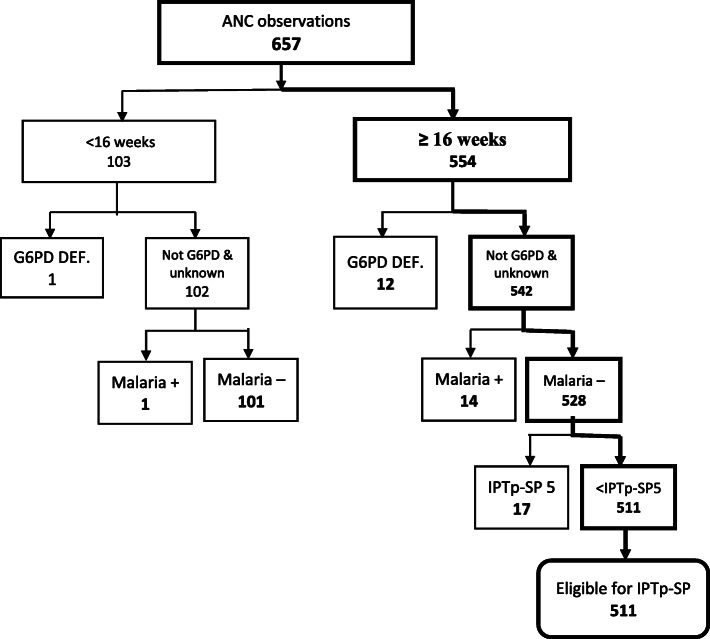
Determining Eligibility of Pregnant Women for IPTp-SP

**Fig. 3:**
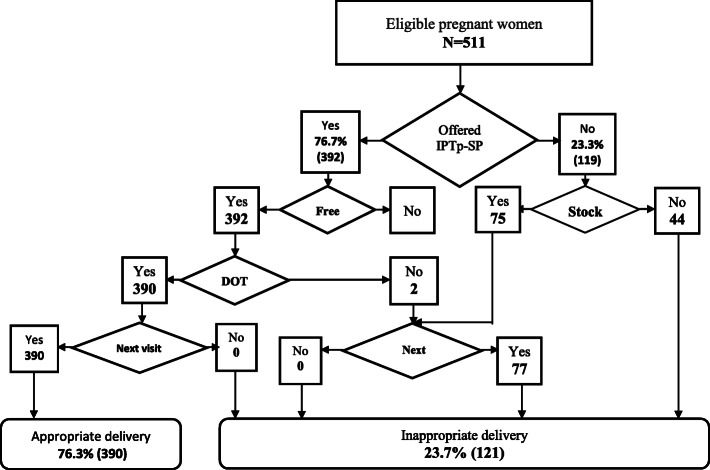
IPTp-SP Delivery Algorithm

**Fig. 4:**
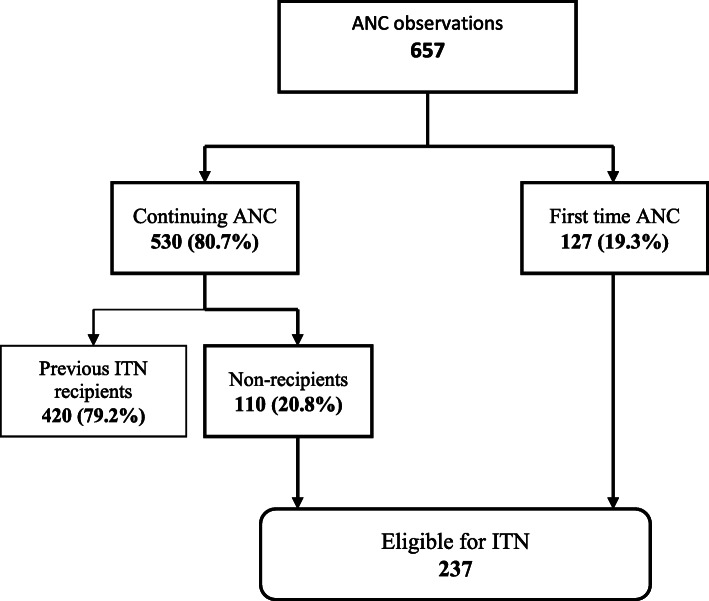
Determining Eligibility of Pregnant Women for ITN

Data were entered and cleaned using Statistical Package for Social Sciences (SPSS) (SPSS Inc., Chicago, IL) version 22, after which the data was imported into STATA 16 SE software (Stata Corp LP) for analysis. Continuous variables in the observation checklist such as gestational age of pregnancy and number of ANC sessions attended were reclassified into ranges. Prior to computing IPTp-SP delivery, ineligible pregnant women (< 16 weeks, G6PD deficient, malaria positive and those who have already taken 5 doses of IPTp-SP) were filtered from the overall sample of pregnant women observed (Fig. 2). This gave rise to a sub-sample of pregnant women, which was then used to assess the delivery of IPTp-SP (Fig. 3). Similarly, ineligible pregnant women (have already received ITN during that pregnancy) were filtered out before the delivery of ITN was computed (Fig. 4). To ensure accurate assessment of the factors influencing the delivery of both IPTp-SP and ITN, inappropriate delivery that occurred during periods of stock out was filtered out, in order not to confound estimation of other predictive variables. Pearson Chi-Square (X^2^) and Fishers Exact Test analysis were used to determine the association between selected explanatory variables and delivery of IPTp-SP and ITN. Statistical significance was set at a *p*-value of less than 0.05 (*p* <  0.05). To ascertain the strength of association between appropriate delivery of the interventions and significant explanatory variables from the X^2^ analysis, a bivariable logistic regression analysis was performed by estimating Odds Ratios (ORs) with 95% confidence intervals (CIs). All explanatory variables with *p* <  0.05 were fitted into an investigator led backward-stepwise multivariable logistic regression model to further ascertain the strength of association with the outcome variable by estimating the adjusted Odds Ratios (aORs). *P*-value of < 0.05 was deemed significant. The results of the study are presented in figures and tables.

### Results determining eligibility of pregnant women for IPTp-SP

Six hundred and eighty observations of the ANC clinic sessions between pregnant women and ANC staff were conducted. Some of the observations had pertinent missing data such as current gestational age, and this was mainly due to unfilled sections in the MHRB. Incomplete records were omitted from the analysis. After data cleaning, 657 (96.6%) of the observed cases had complete datasets tools adequate for analysis. Ineligible women as specified in the Ghana MiP policy document were excluded from the analysis, with the derived sub-sample of pregnant women eligible for IPTp-SP being 511. (Fig. 2).

### Assessment of IPTp-SP delivery

Using the study’s defined delivery assessment algorithm, appropriate and inappropriate delivery of IPTp-SP was computed. Figure 3, shows that out of the 511 pregnant women eligible for IPTp-SP, 76.7% (392) were offered the medication by the ANC staff, of which 390 were administered under DOT, with all of them being told their next ANC visit date. IPTp-SP was appropriately administered to 76.3% of the pregnant women. On the other hand, 23.3% (119) of eligible pregnant women were not offered the medication, 37% (44) of which occurred during a period of stock out. Despite the availability of SP in health facilities during the data collection period, almost 15% (75) of all eligible women were not offered the medication.

### Factors influencing delivery of IPT-SP

Years of experience of the ANC staff was found to be significantly associated (*p* <  0.007) with appropriate delivery of IPTp-SP in the Pearson Chi–Square analysis. Health workers who had 1-5 years experience delivered IPTp-SP appropriately to 88.9% (152) of pregnant women. Type of pregnant woman (first time or continuing ANC client) was also found to be a significant factor (*p* <  0.0001) affecting delivery of IPTp-SP, with 86.1% (360) of the continuing pregnant women having IPTp-SP delivered to them appropriately. The number of ANC sessions attended by pregnant women (*p* <  0.0001) and gestational age of pregnancy (*p* <  0.0001) were also significantly associated with the delivery of IPTp-SP (Table 3).

**Table 3:** Factors Influencing Delivery of IPT-SP

Factors	Delivery of IPTp-SP When in Stock (***N*** = 467)	
	Appropriate	***P***-value
**Type of facility**		
District hospital	96/117 (82.1)	
Faith-based hospital	100/116 (86.2)	< 0.0001
Health Centres	178/205 (86.8)	
CHPS Compounds	16/29 (55.2)	
**Ultrasound scan**		
Available	136/169 (80.5)	
Unavailable	254/298 (85.2)	0.183
**Type of worker**		
Midwife	333/394 (84.5)	
Rotation nurse	57/73 (78.1)	0.173
**ANC years of experience**		
< 1 year	131/171 (76.6)	
1-5 years	152/171 (88.9)	0.007
> 5 years	107/125 (85.6)	
**MiP Training**		
Yes	261/312 (83.7)	0.907
No	129 /155 (83.2)	
**Can SP be taken on an empty stomach**		
Yes (Correct)	107/128 (83.6)	0.977
No (Incorrect)	283/339 (83.5)	
**IPTp-SP can be given at 16 weeks of gestation irrespective of quickening**		
Yes (Correct)	51/67 (76.1)	0.078
No (Incorrect)	339/400 (84.8)	
**Type of Pregnant woman**		
First time ANC	30/49 (61.2)	< 0.0001
Continuing ANC	360/418 (86.1)	
**No. of ANC attended**		
Once	30/49 (61.2)	
Twice	68/77 (88.3)	
Thrice	74/88 (84.1)	< 0.0001
Four	69/82 (84.2)	
Fifth-sixth	90/99 (90.9)	
7+ times	59/72 (81.9)	
**Gestational age**		
16–20 weeks	64/95 (67.4)	
21–25 weeks	71/83 (85.5)	< 0.0001
26–30 weeks	102/110 (92.7)	
31–35 weeks	87/98 (88.8)	
36 + weeks	66/81 (81.5)	

### Determinants of appropriate delivery of IPTp-SP

Factors found to be significantly associated with appropriate delivery of IPTp-SP in the crude analysis were type of health facility, years of experience as an ANC staff, type of pregnant woman, number of ANC clinic sessions attended and gestational age of pregnant woman (Table 4). In the multivariable logistic regression analysis, type of facility, years of experience as an ANC staff and gestational age of pregnant woman were the predictors that remained significant with appropriate delivery of IPTp-SP. Regarding the type of facility, the faith-based hospital (aOR 0.68, 95%CI = 0.275–1.707), the health centres (aOR0.60, 95%CI = 0.236–1.545) and CHPS compounds (aOR 0.12, 95%CI = 0.039–0.375) had reduced odds of appropriate delivery compared to the district hospital. Higher odds of appropriate delivery were found among ANC health workers with one to 5 years’ experience (aOR 3.57, 95%CI = 1.564–8.145) and more than 5 years’ experience (aOR 3.08, 95%CI = 1.181–8.028). The odds of appropriate delivery of IPTp-SP increased with increasing gestational age, where pregnant women with gestational ages between 21 to 25 weeks had more than twice (aOR = 2.33, 95%CI = 1.621–5.343) the odds of appropriate delivery, and those between 26 and 30 weeks had more than four times (aOR 4.64, 95%CI = 1.807–11.918) the odds of appropriate delivery compared to those between 16 to 20 weeks (Table 4).

**Table 4:** Determinants of Appropriate Delivery

Factors	Unadjusted			Adjusted		
	OR	***P***-value	95% CI	aOR	***P***-value	95%CI
**Type of facility**						
District hospital	1.00			1.00		
Faith-based hospital	1.37	0.387	0.6734–2.7756	0.68	0.418	0.2758–1.7075
Health Centres	0.55	0.031	0.3175–0.9479	0.60	0.293	0.2362–1.5452
CHPS Compounds	0.27	0.003	0.1126–0.6432	0.12	0.000	0.0397–0.3752
**ANC years of experience**						
< 1 year	1.00					
1-5 years	2.44	0.003	1.3487–4.4242	3.57	0.002	1.5647–8.1455
> 5 years	1.8	0.056	0.9841–3.3476	3.08	0.021	1.1818–8.0289
**Type of Pregnant woman**						
First time ANC	1.00					
Continuing ANC	3.93	0.000	2.0768–7.4407	–	–	–
**No. of ANC attended**						
Once	1.00					
Twice	4.78	0.001	1.9416–11.7928	–	–	–
Thrice	3.35	0.003	1.4888–7.5271			
Four	3.36	0.004	1.4724–7.6742			
Fifth-sixth	6.33	0.000	2.5897–15.4886			
7+ times	2.87	0.013	1.2519–6.5995			
**Gestational age**						
16–20 weeks	1.00			1.00		
21–25 weeks	2.86	0.006	1.3576–6.0495	2.33	0.044	1.0216–5.3434
26–30 weeks	6.17	0.000	2.6722–14.2728	4.64	0.001	1.8077–11.9186
31–35 weeks	3.83	0.001	1.7919–8.1901	2.73	0.046	1.0158–7.3888
36 + weeks	2.13	0.036	1.0521–4.3170	1.30	0.633	0.4404–3.8469

### Determining eligibility of pregnant women for ITN

Out of the 657 ANC sessions observed, 19.3% (127) of the pregnant women were first time ANC attendees with the remaining being continuing clients. Seventy-nine percent (420) of the continuing clients had “ITN given” documented in their MHRB, with the remaining 21% (110), having no record of ITN given in their MHRB. Defining eligibility for ITN in this study as all pregnant women who have not yet received an ITN, the number of pregnant women eligible for the intervention was 237 as shown in Fig. 4.

### Assessment of ITN delivery

**Fig. 5:**
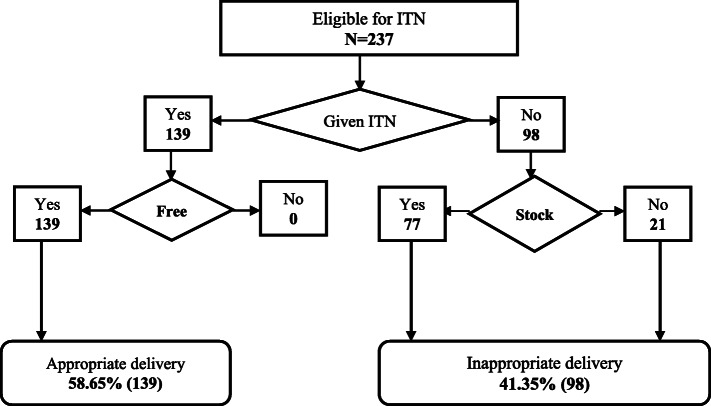
ITN Delivery

Insecticide treated net was appropriately delivered to 58.65% (139) of the 237 pregnant women who had not yet received an ITN prior to day of observation (Fig. 5). Appropriate delivery was highest amongst first time ANC clients (114) than the continuing clients [25] (Table 5). While 21% [21] of inappropriate delivery occurred during periods of stock out, the remaining 32% (77) of eligible women were not given ITN despite stock availability.

**Table 5:** Factors Influencing ITN Delivery

Factors	ITN Delivery When in Stock (***N*** = 216)	
	Appropriate	***P***-value
**Type of facility**		
District hospital	49/69 (71.01)	
Faith-based hospital	32/62 (51.61)	0.102
Health Centres	50/74 (67.57)	
CHPS Compounds	8/11 (72.73)	
**Type of worker**		
Midwife	106/175 (60.57)	0.018
Rotation nurse	33/41 (80.49)	
**ANC years of experience**		
< 1 year	61/95 (64.21)	
1-5 years	42/73 (57.53)	0.149
> 5 years	36/48 (75.00)	
**MiP Training**		
Yes	92/147 (62.59)	0.429
No	47/69 (68.12)	
**Type of Pregnant woman**		
First time ANC	114/117 (97.44)	< 0.000
Continuing ANC	25/99 (25.25)	
**No. of ANC attended**		
Once	114/117 (97.44)	
Twice	20/35 (57.14)	
Thrice	2/22 (9.09)	< 0.000
Four	1/13 (7.69)	
Fifth-sixth	1/11 (9.09)	
7+ times	1/18 (5.56)	
**Gestational age**		
< 15 weeks	64/67 (95.52)	
16–20 weeks	35/49 (71.43)	
21–25 weeks	14/24 (58.33)	< 0.000
26–30 weeks	17/31 (54.84)	
31–35 weeks	7/27 (25.93)	
36 + weeks	2/18 (11.11)	

### Factors associated with ITN delivery

The type of health worker (*p* = 0.018), type of pregnant woman (*p* <  0.0001), number of ANC clinic sessions attended (*p* <  0.0001), and gestational age of the pregnancy (*p* < 0.0001) were significantly associated with delivery of ITN in the Pearson Chi-Square analysis (Table 5). Appropriate delivery of ITN was highest among first time ANC clients (97.44%), compared to continuing clients who have not yet received one (25.25%). Similarly, delivery of ITN decreased with increasing number of ANC attendances.

## Discussion

This study aimed at assessing the delivery of IPTp-SP and ITN to pregnant women attending ANC clinics in the Volta Region of Ghana, using the country’s MiP policy guideline as a guide. The use of structured observation checklists enabled the identification of the intricate delivery practices of the health workers. Furthermore, collection of data across three levels of health care delivery facilities enabled the identification of factors peculiar to each level of care and how it affects the delivery practices of the two preventive interventions.

Stock out of both SP and ITN was one of the health system challenges observed during the data collection period in some facilities and this led to missed opportunities to deliver the interventions to eligible pregnant women. Regarding SP, stock out was common in the health centres, and this could be due to poor quantification of medical commodities. Lower level facilities like the health centres usually do not have personnel like pharmacist or logisticians to manage their medical stores. Hence, other health personnel like nurses or physician assistants take on the task in addition to their clinical duties. If these personnel are not trained on proper quantification of medical commodities to know how to compute their minimum and maximum stock levels, the stock out situation will be inevitable. Similarly, stock out of ITNs were mainly observed in the health centres and this could be attributed to two reasons. The first being the limited medical storage space common to this level of facility, hence, unable to stock adequate quantities because of their bulky nature of ITNs. Secondly, these facilities usually do not have facility owned vehicles to facilitate transportation of medical commodities, therefore delays in the district health directorates transporting requested ITNs regularly could result in constant stockouts.

While IPTp-SP was appropriately delivered to most of the eligible pregnant women in this study, inappropriate delivery was observed across all the facilities, even in the absence of stock out. The unavailability of services such as laboratory and ultrasound scan services in the lower level facilities like the health centres and CHPS compounds could be one of the reasons for inappropriate delivery despite stock availability. This is because starting IPTp-SP is dependent on knowing or ascertaining the gestational age of pregnant women, and in instances where the pregnant woman is uncertain about her gestational age due to poor recall of menstrual periods, the ultrasound scan machine can facilitate that [43]. Also, facilities without laboratory capacity to perform G6PD tests to identify and exclude women who cannot take sulfa containing drugs like SP, could delay the administration of the first dose of IPTp-SP, as pregnant women may be referred to perform these tests elsewhere, and the cost associated with transport and payment of the services could be a barrier, thereby delaying the start of the medication. A study conducted in a health centre in the Dangme West District of Ghana, revealed how pregnant women referred to other facilities for laboratory test discontinued ANC attendance altogether because they could not afford it [44]. Health workforce factors such as forgetfulness [45], poor health worker knowledge on delivery directives [46] and confusion over timing and dosing of SP [47] as shown in other studies could be reasons why the medication was not delivered to eligible pregnant women across all the facilities in this study.

In this study, years of experience of ANC staff was found to be significantly associated with appropriate delivery of IPTp-SP, where workers with more than a years’ experience were three times more likely to deliver IPTp-SP appropriately compared to those with less than a year’s experience. A possible reason could be because ANC staff with less than a years’ experience are newly graduated nurses who have not had the opportunity to attend any training to enlighten them on current approved delivery of IPTp-SP. Training of health workers especially the front line health workers makes them well informed and technically equipped to deliver MiP interventions effectively. However, these trainings are sometimes overlooked thereby affecting their perception about IPTp-SP and how to administer it to pregnant women [48, 49]. A consequence of lack of training on IPTp-SP was reported by Rassi and colleagues [49] where health workers expressed doubts about the efficacy and safety of SP as an IPT because they do not understand why they were asked to discontinue SP for the treatment of clinical malaria, yet asked to retain it as an IPT. These workers indicated that they still use SP to treat symptomatic malaria because they feel it is more efficacious and better tolerated than other available anti-malarial drugs [49]. While a number of studies conducted in other African malaria endemic countries have reported the non-adherence to the DOT strategy of administering IPTp-SP with the most cited reason being unavailability of drinking water [46, 50], this study found the contrary. Almost all the pregnant women who were offered the medication by the ANC staff were given under DOT. Health workers in this context are adhering to the DOT delivery strategy and this could be due to the readily available sachet water in the facilities sold to the women at 20 Ghana pesewas, approximately 0.035 United States Dollars.

The study aimed at identifying gaps in the delivery of IPTp-SP and ITN that needs to be improved to realize optimal uptake of the interventions. Hence, prior to assessing delivery of the interventions, and classifying it as appropriate or inappropriate delivery, ineligible pregnant women as specified by the Ghana MiP policy directive were excluded to limit overestimation of inappropriate delivery as those women may not be given the IPTp-SP or ITN. Additionally, inappropriate delivery during periods of stock out were excluded from the sample before estimating predictors of correct delivery, in order not to confound the estimation of other predictor variables. These are strengths of the study, picked up from a recommendation by Webster and colleagues [47] who acknowledged it as a limitation in their study on assessing delivery effectiveness of IPTp-SP.

### Limitations

There are however a few limitations with this study design. One of such is the possibility of a Hawthorne’s effect [51] on the ANC health staff. Acknowledging the fact that the observed staff may modify their behavior in response to their awareness of being observed, in this case gravitating towards ‘appropriate behaviour’, the observations were conducted over a number of days in the study facilities. Therefore, these health workers are likely to revert to their normal delivery practices, thereby enabling the study to assess the true delivery practices. It is important to note that, the Hawthorne’s effect may be applicable in a context where the health workers are aware of the appropriate delivery practices but do not practice it, therefore, they would modify their behavior when they become conscious of observers. On the other hand, if the health workers are not aware of the appropriate delivery practices, being observed or not, will not have any effect on their delivery practices. Another, limitation of this study could be the inability to capture all the delivery practices of the ANC staff by the observing research assistants. This is because the pace of delivery is not determined by the RAs, and this pace could be exceptionally swift on days where the ANC attendance is high. However, to curtail this limitation, community health nurses who are familiar with the health care delivery setup and more inclined to understand the clinical care and medical jargons were recruited as research assistants. Furthermore, rigorous training on data collection tools, coupled with extensive pre-test activities were carried out prior to the actual data collection to ensure that the RAs are able to collect the data accurately by looking and listening attentively to the delivery of care by ANC health staff.

## Conclusions

In conclusion, inappropriate delivery of IPTp-SP was observed across all three levels of the health care delivery facilities, and predictors of appropriate delivery were type of facility, years of experience of ANC staff and gestational age of pregnant woman. It should be noted that in the absence of stock as observed in mainly the hospitals, eligible pregnant women missed out on being given the medication. This highlights the facts that there could be other underlining factors which might be better elucidated through qualitative means of enquiry such as in-depth interview of health workers. Inappropriate delivery of ITN was also observed across all the health facilities included in this study.

## Data Availability

The dataset analyzed for this study is available from the corresponding author on reasonable request.

## Abbreviations

ANC: Antenatal Care
CHPS: Community-Based Health Planning Services
DHIMS: District Health Management Information System
DOT: Directly Observed Therapy
GDHS: Ghana Demographic and Health Survey
GFR: Genaral Fertility Rate
IPTp-SP: Intermittent Preventive Treatment for Pregnant women using Sulfadoxine Pyrimethamine
ITNs: Insecticide Treated Nets
MHRB: Maternal Health Record Book
MiP: Malaria in Pregnancy
*P.*: *Plasmodium*
POC: Point of Care
SP: Sulfadoxine Pyrimethamine
SSA: Sub-Saharan Africa
WHO: World Health Organization
